# The Only way is up? How Different Facets of Employee and Supervisor Perfectionism Help or Hinder Career Development

**DOI:** 10.1177/00332941241229204

**Published:** 2024-01-29

**Authors:** Kathleen Otto, Martin Baluku, Amelie Schaible, Cemre Oflu, Emily Kleszewski

**Affiliations:** Department of Work and Organizational Psychology, 9377 Philipps University of Marburg, Marburg, Germany; School of Psychology, 58588 Makerere University, Kampala, Uganda; Department of Work and Organizational Psychology, 9377 Philipps University of Marburg, Marburg, Germany

**Keywords:** multidimensional perfectionism, career, intrinsic success, work motivation, self-determination theory, leadership, delegation, illegitimate tasks

## Abstract

Although the double-edged nature of perfectionism is widely acknowledged, little is known about how it shapes employee career development. By combining two field studies, we provide a multiperspective insight into the relevance of both employee and supervisor perfectionism for employee career development. While we expected self-oriented perfectionism (SOP) to have an ambivalent role for career development, we proposed that socially prescribed perfectionism (SPP) in particular, but also other-oriented perfectionism (OOP), would show maladaptive relationships with career-related indicators. In Study 1 (*N* = 116), we focused on the employee perspective and how multidimensional perfectionism relates to career aspirations (operationalized via work motivation) and subjective career success. Employees high in SOP reported higher, whereas those high in SPP reported lower perceived career success. OOP was negatively related to intrinsic motivation, but positively explained extrinsic (social) motivation and amotivation. In Study 2 (*N* = 146), we examined the role of supervisor perfectionism in supporting or hindering employees’ career development by providing or draining resources. Our results show that supervisors high in SOP - and partly in OOP are reluctant to delegate highly responsible tasks; SPP even increased the likelihood of assigning illegitimate tasks to subordinates. Our findings suggest that both employee and supervisor perfectionism may boost or thwart employee career development and success. We discuss that supervisor perfectionism may limit employees’ opportunities for experiential learning.

## Introduction

When entering the application phase, career starters frequently get the advice to answer the question about their biggest weakness in a job interview by stating that they are perfectionists. Yet why should this seemingly weakness turn out to ease job entry and career progress? Obviously, applicants assume that perfectionism is a “most wanted” trait leading firms to preferably hiring people with such a personality. Avoiding mistakes, trying to achieve best results and performing flawlessly, that is, being a perfectionist seems to be a safe investment into a successful career. But does perfectionism really boost one’s career, or is it a drain on important resources that employees can no longer invest in advancing their careers? With this study, we look at the role of perfectionism for employees’ career from the lens of conservation of resources theory (Hobfoll, 1989; Hobfoll et al., 2018), and combine two perspectives. In Study 1, we shed light on how perfectionist employees differ in work motivation as a career-relevant resource (Haenggli & Hirschi, 2020) and in perceived career success. In Study 2, we focus on how multidimensional perfectionism in supervisors helps or hinders employees’ career development by supporting employees or by draining their resources (e.g., by being given tasks that develop skills or are seen as illegitimate).

In their meta-analysis, Ng and colleagues considered four sets of predictors, namely human capital, organizational sponsorship, socio-demographic status, and stable individual differences, as relevant antecedents of career success (Ng et al., 2005). Building on this framework, we particularly focus on the fourth set of predictors by introducing perfectionism as a so far neglected but important individual difference concept impacting employees’ career development. Already Slaney and colleagues mentioned in their paper more than 25 years ago that the impact of perfectionism on career success should be investigated (Slaney et al., 1995). While in the meantime some studies have linked multidimensional perfectionism with career adaptability, career decision-making, and career search self-efficacy (e.g., Ganske & Ashby, 2007; Gnilka & Novakovic, 2017; Stoeber et al., 2016), career success itself has been largely ignored so far (for an exception, see Kundi et al., 2021).

Ng and colleagues further link organizational sponsorship with career success. The dimension of organizational sponsorship contains facets of supervisory support and access to skill development. Obviously, leaders play a prominent role in designing career-promoting work through providing opportunities to advance one’s skills. We further assume that the degree of how much an employee receives work that enhances or hinders career opportunities depends on the perfectionism of the leader. While leader perfectionism and its consequences for leadership behavior over all is still in its infancy (Guo et al., 2020; Otto et al., 2021) the knowledge about how perfectionistic leaders shape employees’ career specifically is completely unexplored.

Besides the paucity of research linking perfectionism to career development, studies on perfectionism in the career-related context nearly exclusively investigated student samples, be it either secondary school students or university students, yet careers develop throughout one’s working life (Nagy et al., 2019). Research has so far ignored people further ahead in career (see Kundi et al., 2021, for an exception). The present study therefore contributes to previous research by investigating how employee and supervisor perfectionism may boost or constrain career success. While various operationalizations of multidimensional perfectionism were applied in previous research none of the studies examined how perfectionistic expectations towards others (i.e. other-oriented perfectionism) shape career development.

In sum, we seek to develop and extend the limited literature on perfectionism and career by (a) investigating aspirations and perceived career success in a sample of professionally experienced employees, (b) by considering a broader conceptualization of multidimensional perfectionism as in past research, and (c) by exploring the role of supervisor perfectionism combining an employee with a leader perspective. In the following we will first define the concept of perfectionism in detail before introducing our present research.

### Multidimensional perfectionism

Perfectionism as a personality trait has been of central interest in research for a long time and has gained even more attention in recent years, especially in the workplace context (Ocampo et al., 2020). It is commonly understood as attempting to reach flawlessness and setting unrealistically high standards for performance accompanied by tendencies for overly critical evaluations (Hewitt & Flett, 1991; Frost et al., 1990). Perfectionism is a multidimensional construct and its dimensions can be differently related to maladaptive or adaptive behavior and individual functioning in employees (Harari et al., 2018; Ocampo et al., 2020). One of the most commonly researched and influential models regarding the multiple dimensions of perfectionism is Hewitt and Flett’s (1991) tripartite model of perfectionism.

In their model, Hewitt and Flett (1991) differentiate three dimensions of perfectionism: self-oriented perfectionism, socially prescribed perfectionism, and other-oriented perfectionism. The difference between these dimensions of perfectionism lies within the focus of the perfectionistic behavior (self-oriented vs. other-oriented perfectionism), or the origin of perfectionistic demands (socially prescribed perfectionism).

According to Hewitt and Flett (1991), self-oriented perfectionism refers to perfectionism that is directed at the self. It comprises characteristics such as setting extraordinarily high standards for oneself and striving for exceedingly high-performance goals. Socially prescribed perfectionism is characterized by the belief that a demand for perfectionism and unrealistically high standards are set by significant others. It comprises characteristics such as the need to be perfect in order to be liked by others or to be seen as worthy, and the constant perception of high social pressure (Hewitt & Flett, 1991). Other-oriented perfectionism, by contrast, describes exceedingly high standards that are directed towards significant others. Other-oriented perfectionists demand perfection from others, highly value other people being perfect, and evaluate others’ performance with regards to their own standards. If they do not meet these standards, others easily get blamed, as other-oriented perfectionism is also characterized by limited trust in others and feelings of hostility towards them (Hewitt & Flett, 1991; Stoeber, 2014).

Over time, different models with different dimensions of perfectionism have been proposed (Frost et al., 1990; Slaney et al., 2001). These models have in common that the proposed dimensions can be organized in two overarching and interrelated factors. These factors can be referred to as perfectionistic strivings, comprising high performance standards, and perfectionistic concerns, encompassing concern over mistakes and the fear of negative evaluation (for a comprehensive review, see Stoeber & Otto, 2006). Self-oriented perfectionism and socially prescribed perfectionism are key indicators of perfectionistic strivings and perfectionistic concerns (e.g., Stoeber & Damian, 2016). A large body of research demonstrated that perfectionistic concerns and associated dimensions usually show maladaptive associations with outcomes such as employees’ stress, motivation, and work engagement (e.g., Harari et al., 2018; Stoeber et al., 2016). Perfectionistic strivings, by contrast, may even show adaptive relationships with employees’ motivation and work engagement, in particular, once the overlap between the dimensions is accounted for. However, the role of other-oriented perfectionism within this superordinate factors remains unclear as researchers often focus on the key indicators of perfectionism. Thus, this dimension has been labelled as an “other form” of perfectionism (Ocampo et al., 2020). As opposed to the other dimensions proposed by Hewitt and Flett (1991), other-oriented perfectionism is also uniquely associated with personality traits that form the so called “dark triad”, narcissism, Machiavellianism, and subclinical psychopathy (Stoeber, 2014). Thus, other-oriented perfectionists are likely to be unsupportive and uninterested in another people’s well-being.

### Investigating the role of employee and supervisor perfectionism from the lens of conservation of resources theory

The conservation of resources (COR) theory (Hobfoll, 1989; Hobfoll et al., 2018) posits as a basic tenet that humans are motivated to protect their current resources and to acquire new resources. Resources are valuable for employees because they support goal attainment (Halbesleben et al., 2014). Subjective and objective career success are personally important goals and employees have to use resources (e.g., motivation and effort) to advance their career development (Haenggli & Hirschi, 2020). However, resources are finite and employees have to make decisions about where to allocate their resources in order to acquire resources, protect themselves from resource loss, and attain their goals (Halbesleben et al., 2014; Kaplan & Gangestad, 2005). Studies underscore that employees differ in their allocation decisions at work (e.g., Penney et al., 2011). We argue that some allocation decisions are helpful for career advancement, while others may hinder it. In addition to their own role in shaping career development, employees may be situated in more or less supportive work contexts. For example, they may have supervisory support and access to skills development (Ng et al., 2005) that provide them with career-relevant resources, or they may face career hurdles that deplete their resources (Ng & Feldman, 2014). Based on these ideas, we argue that the different dimensions of employee perfectionism are related to different decisions about where to allocate resources, whereas supervisor perfectionism may be a source of career hurdles for employees and thus impede their career success.

## Study 1: employee perfectionism, work motivation and perceived career success

In Study 1, we aimed to investigate how the dimensions of perfectionism, including other-oriented perfectionism, shape employees’ career aspirations (i.e., work motivation) and career success. We focus on motivation because not only are the dimensions of perfectionism characterized by different motivational qualities (Stoeber et al., 2018), but also work motivation is a meaningful resource for employees’ careers (Haenggli & Hirschi, 2020).

Studies based on self-determination theory (SDT) support the idea that there are two kinds of aspirations, which people try to achieve: extrinsic and intrinsic aspirations (Niemiec et al., 2009). Mirroring these distinctions in case of career success, there are two broad dimensions (Arthur et al., 2005) one describing extrinsic career success characterized by verifiable indicators (e.g., position), and the other called intrinsic career success that represents individuals’ subjective perceptions of their career achievements. Hall and Chandler state that “true success is not just getting what you want in life – it’s liking what you get” (2005, p. 19). Intrinsic or subjective career success tends to be more relevant than extrinsic career success (Sobiraj et al., 2016) and can even be regarded as a base for achieving higher extrinsic career success in the long run (Otto et al., 2017). Accordingly, we focus on linking employee perfectionism with *intrinsic career success*, which reflects the subjective evaluation of how much people are satisfied with their professional development (Judge et al., 1995).

Furthermore, SDT differentiates between various reasons or motivators to attain these aspirations (Deci & Ryan, 1985; Ryan & Deci, 2000). Specifically, SDT presumes that individuals strive to achieve extrinsic or intrinsic goals or both; and particularly, the intrinsic aspirations are related to the psychological need for competence (Ryan & Deci, 2000). As these strivings are reflected in work motivation, above intrinsic career success we explore the role of employee perfectionism for various facets of *work motivation*, namely amotivation, extrinsic motivation (facets: social and material), and intrinsic motivation.

Previous research on perfectionism and motivation has predominantly been conducted with students and athletes and focused on the key indicators of perfectionism. This research revealed different motivational qualities inherent in the dimensions of perfectionism, such that perfectionistic strivings are characterized by motivations and regulatory styles that include higher degrees of self-determination such as intrinsic motivation, integrated regulation, and identified regulation (see Stoeber et al., 2013; for a study on perfectionism and work motivation). Perfectionistic concerns, by contrast, are characterized by motivations and regulatory styles that include lower degrees of self-determination such as amotivation, external regulation, and introjected regulation (Stoeber et al., 2018). In line with this, research has linked self-oriented perfectionism to internal motivation and socially prescribed perfectionism to external motivation in students (e.g. Stoeber et al., 2009).

In addition, the dimensions of perfectionism should relate to different decisions on where to allocate resources such as attention, time, and effort. Based on the dual process model of perfectionism (Slade & Owens, 1998), self-oriented perfectionists are generally driven by approach behavior and in that pursue perfection, success, and approval as goals. Such employees should challenge themselves to approach and achieve their goals at work, which facilitates competence satisfaction (Kleszewski & Otto, 2023), seeking opportunities to grow, and insofar career development. Research underscore that they show high effort in achieving their goals (Hrabluik et al., 2012). In contrast, socially prescribed perfectionists are driven by avoidance behavior and, while avoiding imperfection, are constantly confronted with their own inadequacy and anxiety about not meeting the expectations of supervisors, colleagues, and clients. Studies support that much attentional resources of those employees are drained by worrying and ruminating (e.g., Flaxman et al., 2012), which is why resources for advancing career may lack. This avoidance and lack of confidence can be seen as counterproductive with regard to one’s abilities and career development. In support of our arguments, research has linked self-oriented perfectionism to mastery and self-efficacy, and socially prescribed perfectionism to perceptions of task failure and low self-efficacy (Mills & Blankstein, 2000; Stoeber et al., 2015; Stoeber & Yang, 2010). Above recent research (Pannhausen et al., 2022) emphasizes the lack of confidence that individuals high in socially prescribed perfectionism have in their own abilities and linked this dimension, but not the dimension of self-oriented perfectionism, to the impostor phenomenon. Other-oriented perfectionism, by contrast was negatively related to the impostor phenomenon.

Prior research on perfectionism and career has primarily focused on adolescents and young adults such as students ahead of graduation and points towards different attitudes towards career planning and development inherent in the dimensions of perfectionism. In these samples, perfectionistic strivings have been positively related to career search efficacy and career decision-making self-efficacy, and negatively related to the perception of career barriers (Ganske & Ashby, 2007; Gnilka & Novakovic, 2017). Perfectionistic concerns, on the contrary, have been found to relate to negative career thinking, low career search and career decision-making self-efficacy, the presence of career barriers, and career indecision (Andrews et al., 2014; Ganske & Ashby, 2007; Gnilka & Novakovic, 2017; Lehmann & Kostam, 2011). In another study with university students, Stoeber et al. (2016) found self-oriented perfectionism to display positive associations with career adaptability and career optimism, whereas they found a negative association between socially prescribed perfectionism and career adaptability. They also included other-oriented perfectionism and found this dimension to relate positively with perceived knowledge of the job market. So far, only one study (Kundi et al., 2021) investigated perfectionism and career-related outcomes in employees. This study found perfectionistic strivings to show positive correlations with career adaptability, career satisfaction and career commitment. However, the authors did not measure perfectionistic concerns.

Apart from the need to investigate perfectionism, motivation and career success in employees, it remains to be investigated whether the focus on others inherent in other-oriented perfectionism may consume important resources and distract employees high on this dimension from pursuing their own career goals. In particular, researchers highlighted the need to investigate the role of this “other form” of perfectionism concerning motivation (Stoeber et al., 2018).

Against this background on different motivational qualities, different attitudes on career planning in students and findings from previous studies, we expect that the dimensions of perfectionism relate differently to motivation and career success. Specifically, self-oriented perfectionism is considered as rather adaptive regarding motivation and career success, whereas a maladaptive role is attributed to socially prescribed perfectionism. Concerning other-oriented perfectionism with its focus on others, we expect this dimension to display a lack of intrinsic motivation. Instead, employees high on this dimension may be externally motivated and even show a tendency towards amotivation. Their constant focus on others performance and mistakes may also distract them from concentrating on their own goals and career development. Nevertheless, research has linked this dimension to knowledge of the job market (Stoeber et al., 2016) and to dominance and leadership goals (Stoeber, 2014) which may point towards assertiveness. Thus, the association of other-oriented perfectionism and career success will be regarded on an exploratory basis.

H1: Employee self-oriented perfectionism is (H1a) positively related to intrinsic motivation and is further (H1b) positively linked with perceived career success.

H2: Employee socially prescribed perfectionism is (H2a) positively related to extrinsic motivation and (H2b) negatively related to perceived career success.

H3: Employee other-oriented perfectionism is positively related to (H3a) extrinsic motivation and (H3b) amotivation.

## Method

### Participants

Administrative staff of a German university received invitations to complete an online survey on career success through the internal email distribution service. Precondition for participations was to have a working contract of at least 20 hours a week. Overall, *N* = 116 employees participated of which 81.9% were female, while 18.1% identified as male. The participants’ ages ranging from 19 to 65 years (*M* = 43.67, *SD* = 11.28). On average, they had been working in their current professions for 12.84 years (*SD* = 11.10), and the mean for their average weakly working hours was 36.66 (*SD* = 8.17).

### Instruments

#### Perfectionism

All participants completed the 15-item German revised short form of the Multidimensional Perfectionism Scale (HF-MPS, Hewitt & Flett, 1991; German translation: Altstötter-Gleich, 1998). Following Stoeber (2018), items of the short form proposed by Cox et al. (2002) were used to assess self-oriented perfectionism (5 items; e.g., “One of my goals is to be perfect in everything I do.”) and socially prescribed perfectionism (5 items; e.g., “People expect nothing less than perfection from me.”). In addition, the short form introduced by Hewitt et al. (2008) was used to measure other-oriented perfectionism (5 items; e.g., “Everything that others do must be of top-notch quality.”). The items were presented with the HF-MPS’s standard instruction in German (“Listed below are a number of statements concerning personal characteristics and traits…“) and answered on a 7-point Likert varying from “disagree” (1) to “agree” (7). When tested for reliability, the HF-MPS subscales showed good Cronbach’s alpha values for self-oriented perfectionism α = .82; other-oriented perfectionism α = .80; socially prescribed perfectionism α = .85).

#### Work motivation

*To reflect various facets of work motivation we applied the Multidimensional Work Motivation scale (*Gagné et al., 2015). Specifically, we provided the participants with the question *“Why do you or would you put efforts into your current job?” and asked them to answer it on the following four dimensions*: amotivation *(3 items; “*I don’t, because I really feel that I’m wasting my time at work.”), extrinsic social motivation (3 items; “To avoid being criticized by others (e.g., supervisor, colleagues, family, clients …”), extrinsic material motivation (3 items; “Because others will reward me financially only if I put enough effort in my job (e.g., employer, supervisor …“), and intrinsic motivation (3 items; “Because what I do in my work is exciting.“). Answers had to be given on a *scale from “not at all” (1) to “completely” (6). The measures showed satisfactory to excellent* Cronbach’s alpha values, that is, a*motivation:* α = .96, extrinsic social motivation: α = .85, extrinsic material motivation: α = .74, and intrinsic motivation: α = .95.

#### Perceived career success

Career satisfaction was assessed with the German version (Abele & Spurk, 2009) of the Career Satisfaction Scale (CSS, Greenhaus et al., 1990; 5 items; e.g. “I am satisfied with the progress I have made toward meeting my goals for income”). The scale had to be answered on 6-point Likert scale and had a Cronbach’s α of .90. In addition, we used the self- and other-referent career success measure by Heslin (2003) which was shortened to one item for the two subscales of self-referent success (i.e., “I am satisfied with the overall success I have achieved in my career relative to my career aspirations.“), and other-referent success (i.e., “I am satisfied with the overall success I have achieved in my career relative to my career peers.“).

## Results and discussion

As shown in Table 1, and in accordance with previous findings (e.g., Stoeber, 2014; Stoeber et al., 2017), the three perfectionism facets were significantly positively correlated indicating substantial overlap. Also, the dependent variables, that is, the work motivation facets and the perceived career success indicators, revealed some to be expected relationships. Specifically, intrinsic motivation was negatively associated with amotivation and extrinsic social motivation, while both extrinsic motivation facets were positively linked as well as amotivation with extrinsic social motivation. All perceived career success indicators were highly correlated with each other.

**Table 1.** Correlations and Descriptive Statistics (Study 1, Employees in the university administration).

Variable	1	2	3	4	5	6	7	8	9	10	11	12	13
1. Gender													
2. Age	−.10												
3. Professional tenure	−.06	.70***											
4. Self-oriented perfectionism	−.23*	−.12	−.05										
5. Socially prescribed perfectionism	−.10	−.18	−.24*	.39***									
6. Other-oriented perfectionism	−.03	−.06	−.12	.33***	.24*								
7. Amotivation	.12	−.13	−.03	.05	.12	.20*							
8. Extrinsic social motivation	−.19*	−.31**	−.37***	.30**	.27**	.33***	.28**						
9. Extrinsic material motivation	.01	−.46***	−.38***	.24**	.21*	.15	.13	.43***					
10. Intrinsic motivation	.02	.03	−.02	.03	−.12	−.23*	−.70**	−.29**	−.05				
11. Career satisfaction	.06	.00	−00	.11	−.20*	.06	−.43***	−.03	−.05	.40***			
12. Other-referent career success	−.01	.00	.02	−.03	−.29***	.05	−.50***	−.08	−.01	.41***	.77***		
13. Self-referent career success	.03	.01	.04	.01	−.25**	.06	−.51***	.01	.04	.43***	.78***	.85***	
*M*	——	43.85	12.87	3.87	2.59	3.25	1.84	3.65	2.69	4.55	3.93	4.02	4.03
*SD*	——	11.34	11.10	0.85	0.90	0.83	1.16	1.16	1.13	1.23	1.10	1.33	1.41
Cronbach’s alpha	——	——	——	.82	.85	.80	.96	.85	.74	.95	.90	——	——

*Note. N* = 116. For gender, 1 = female, 2 = male. Professional tenure reflects the years in the practiced profession. Scores are mean scores with a possible range of 1–6 with 6 indicating strong endorsement to the construct.

*p < .05. **p < .01. ***p < .001.

We used hierarchical regression analyses to adequately test our hypotheses and shed light on the unique relationships of each perfectionism facet when the other two facets were simultaneously accounted for. Because several studies evidenced that career success strongly depends on gender, age, and professional experiences (Judge et al., 1995), these three socio-demographic variables were considered as relevant control variables in the first step before the three perfectionism dimensions were included in the second step of the analyses.

As illustrated for the relationship of the perfectionism facets and work motivation in Table 2, *employee self-oriented perfectionism* was not substantially related to intrinsic motivation contradicting our assumptions in H1a. This finding is inconsistent with previous studies conducted in employees and students (e.g., Stoeber et al., 2009, 2013). Unexpectedly yet self-oriented perfectionists reported higher extrinsic material motivation. While this at first sight seems to be a surprising finding it might be explained by the fact that salary or income is a central parameter of extrinsic or objective career success (Arthur et al., 2005). We like to cautiously speculate that the aspiration to aim high and to make progress in one’s career is reflected by this positive link of self-oriented perfectionism and extrinsic material motivation. This finding also aligns with the fact that individuals high on this dimension pursue success and approval as goals (Slade & Owens, 1998). Further in Table 3 the associations of perfectionism with career success are reported. In line with our predictions, employee self-oriented perfectionism was positively linked to other-referent career success supporting H1b. Specifically, self-oriented perfectionist employees evaluated that compared to their peers they had achieved more in their career so far.

**Table 2.** Hierarchical Regression Analyses: Predicting Work Motivation by Employees’ Perfectionism (Study 1).

	*B*	*SE*	B	*CI 95%*	*R* ^*2*^ *change*	*B*	*SE*	β	*CI 95%*	*R* ^*2*^ *change*
	Amotivation					Extrinsic social motivation				
Step 1	1.50				.043	2.55				.176***
Gender	0.37	.28	.13	[–.232, 1.04]		−0.39	.26	−.13	[–.870, .060]	
Age	−0.02	.01	−.21	[–.045, .004]		−0.01	.01	−.10	[–.028, .010]	
Professional tenure	0.01	.01	.12	[–.018, .041]		−0.03	.01	−.26*	[–.054, .000]	
Step 2					.049					.105**
Self-oriented perfectionism	−0.05	.14	−.04	[–.359, .231]		0.19	.13	.14	[–.105, .444]	
Socially prescribed perfectionism	0.12	.13	.10	[–.188, .460]		0.09	.12	.07	[–.168, .383]	
Other-oriented perfectionism	0.26	.13	.20*	[–.109, .581]		0.31	.12	.22*	[–.020, .584]	
	Extrinsic material motivation					Intrinsic motivation				
Step 1	3.08				.219***	5.35				.003
Gender	0.08	.26	.03	[–.468, .606]		−0.02	.30	−.01	[–.654, .578]	
Age	−0.03	.01	−.35**	[–.054, −.013]		0.01	.01	.09	[–.019, .042]	
Professional tenure	−0.01	.01	−.12	[–.033, .010]		−0.01	.01	−.12	[–.042, .018]	
Step 2					.043					.069✝
Self-oriented perfectionism	0.23	.13	.17✝	[–.080, .525]		0.13	.15	.09	[–.148, .438]	
Socially prescribed perfectionism	0.06	.12	.05	[–.168, .308]		−0.15	.14	−.11	[–.483, .193]	
Other-oriented perfectionism	0.07	.12	.05	[–.178, .289]		−0.34	.14	−.24*	[–.649, .003]	

*Note.* For details, see Table 1. CI = confidence interval. Confidence intervals rely on 5000 bias-corrected and accelerated bootstrap-samples.

✝ *p < .10. *p < .05. **p < .01. ***p < .001, two-tailed tests.

**Table 3.** Hierarchical Regression Analyses: Predicting Perceived Career Success by Employees’ Perfectionism (Study 1).

	*B*	*SE*	B	*CI* 95%	*R* ^2^_change_	*B*	*SE*	B	*CI* 95%	*R* ^2^_change_
	Career satisfaction					Other-referent career success				
Step 1	4.02				.010	3.27				.012
Gender	0.12	.27	.04	[–.405, 689]		0.43	.31	.13	[–.097, .997]	
Age	−0.003	.01	−.03	[–.023, .020]		0.01	.01	.05	[–.023, .034]	
Professional tenure	0.004	.01	.04	[–.021, .029]		−0.01	.02	−.04	[–.035, .023]	
Step 2					.079*					.087*
Self-oriented perfectionism	0.13	.13	.10	[–.149, .417]		0.36	.16	.24*	[.015, .725]	
Socially prescribed perfectionism	−0.36	.12	−.31**	[–.603, −.110]		−0.41	.15	−.29**	[–.686, −.115]	
Other-oriented perfectionism	0.13	.13	.10	[–120, .355]		0.08	.15	.05	[–.243, .355]	
	Self-referent career success									
Step 1	4.51				.004					
Gender	0.02	.34	.01	[–.551, .626]						
Age	−0.003	.02	−.02	[–.032, .027]						
Professional tenure	0.001	.02	.01	[–.029, .032]						
Step 2					.100**					
Self-oriented perfectionism	0.12	.17	.07	[–.194, .458]						
Socially prescribed perfectionism	−0.52	.16	−.35***	[–.830, −.211]						
Other-oriented perfectionism	0.18	.16	.11	[–.125, .450]						

*Note.* For details, see Table 1. CI = confidence interval relying on 5000 bias-corrected and accelerated bootstrap-samples.

*p < .05. **p < .01. ***p < .001.

Next, *employee socially prescribed perfectionism* displayed significant bivariate correlations with extrinsic social and material motivation. However, these associations were not significant in the regression analyses when the overlap with self-oriented and other-oriented perfectionism were accounted for, leading us to reject H1b which assumed a positive link of this perfectionism dimension with extrinsic work motivation. Mirroring the finding of self-oriented perfectionism and intrinsic motivation above, these results are inconsistent with findings from previous studies conducted in students (Stoeber et al., 2009). Yet, socially prescribed perfectionism was negatively related to all three facets of career success. As proposed in H2b, socially prescribed perfectionists perceived lower career satisfaction as well as evaluated their own career achievements to be lower when either compared to their own aspirations (self-referent career success) or to career peers (other referent career success). These findings align with the already negative association between socially prescribed perfectionism and attitudes on career planning among students (e.g., Stoeber et al., 2016), and provide evidence that these attitudes may translate into reduced career success in employees working life.

Finally, we assumed *other-oriented perfectionist employees* to report higher extrinsic and amotivation. We found other-oriented perfectionism to predict extrinsic social motivation and amotivation providing support of our theorizing in H3a and H3b. Moreover, unexpectedly, it further was negatively linked to intrinsic motivation. However, while the regression coefficients indicated significant relationships, the confidence intervals of the bootstrapped resamples did not (see Table 2), suggesting that overall the results for other-oriented perfectionism should be treated with caution. Nevertheless, they highlight the potential of examining this “other form” of perfectionism in relation to motivation. Although the demands of other-oriented perfectionism are directed towards others, this external focus may be highly relevant for employees’ own motivation and even lead to a lack of intrinsic motivation. It may be speculated that the focus on others inherent in this dimension may distract employees from pursuing their own goals and fully investing themselves into their job.

## Study 2: Supervisor perfectionism and employee career development

In addition to employees’ own perfectionism, supervisor perfectionism may be relevant for employees’ career development opportunities or hurdles, which is why we aim to address this question in Study 2. As supported by meta-analytic evidence, Ng et al. (2005) found organizational sponsorship – including such facets as supervisory support and access to skill development – as relevant predictors for career success. Obviously, supervisors’ behavior at their workplace influences the well-being of their employees, as well as the efficiency of their department, company, etc. (e.g., Bowers & Seashore, 1966; Gregersen et al., 2014; McAlearney, 2008). While there is still not much research to date, a link between supervisor perfectionism and the executed leadership behavior has recently been established (Guo et al., 2020; Otto et al., 2021). From the perspective of COR theory, perfectionistic supervisors may drain important resources from their employees. These resources may not be available for employees to acquire new resources (e.g., skills) and advance their careers. A recent study underscores that perfectionistic supervisors drain resources from their employees (Malik, 2023).

Answering the question about the role of perfectionism for employees’ career development, it seems important to acquire a deeper insight on how perfectionistic supervisors shape their work environment and treat their employees. Do they allow their subordinates the freedom to develop their skills? Are they able to delegate such tasks that promote career development and for which fulfilment their subordinates are completely in charge? Do they preferably delegate low-promotility tasks, that is, such that might be perceived by subordinates as a threat to their professional identity?

There are many theories regarding effective leadership behavior, focusing on different aspects. Yukl (2013) proposed that managers are most effective if they use participative leadership and thereby allow their subordinates to have influence on the leaders’ decision. Decision-making can be seen as a central aspect of leadership. Making sensible decisions that benefit the organization and are supported by their subordinates is an important function of a leader. The process of leaders’ decision-making and especially the extent to which it is shared with the leaders’ subordinates has been of great interest in past research (Heller & Yukl, 1969; Vroom & Jago, 1973). Many different taxonomies address the forms of participation in decision-making, but the most widely accepted is that of participation as a continuum ranging from autocratic decision-making (leader makes own decision without explanation) to delegation (subordinate is fully in charge of decision; Heller & Yukl, 1969; Vroom & Yetton, 1973). The desired benefits of joint decision-making are diverse and include improved decision quality, greater acceptance and understanding of decision by all participating parties, and development of subordinates’ decision-making skills (Yukl, 2013).

Delegation is commonly seen as the most participative management technique (Vroom & Yetton, 1973). It is defined as the manager allowing an individual or a group of subordinates to make decisions by themselves and to uphold responsibility for their decisions. Delegation is seen as the direct opposite of autocratic decision-making, increasing subordinates’ latitude and giving them more freedom of choice on how to do their work (Heller & Yukl, 1969). The supervisor might still hold responsibility for setting a supportive framework and to specify certain conditions regarding results, but the overall decision-making process lies with the subordinates (Yukl, 2013). Delegation is an often-recommended aspect of leadership behavior. The purpose of delegation can differ considerably among supervisors and situational circumstances. Yukl and Fu (1999) found the main reasons for managers to delegate to be the development of their subordinates’ skills, confidence, and problem-solving abilities, to increase commitment and to make their employees’ job more interesting.

Regarding positive effects of delegation on employee work outcomes, Xiong Chen and Aryee (2007) found delegation to be related to affective organizational commitment and innovative behavior. So far, there have been no publications on how supervisors’ personality may shape the usage of delegation. Also, no studies so far have looked at different aspects of delegation. Delegated tasks ought to differ with respect to the amount of responsibility and latitude that is permitted. Consequently, it seems crucial to explore a broader picture of this leadership behavior. The delegation of certain tasks might even have negative effects on employees, for example if they perceive them as being illegitimate.

Specifically, task assignments can be seen as illegitimate by employees if they either perceive them as being unreasonable or unnecessary (Semmer et al., 2015). Unreasonable tasks refer to assignments that are not inherent in an employee’s professional role, for example if a professional with high working experience is supposed to do a novice’s work. Contrary, tasks can be seen as unnecessary if they seem inefficient, for example because they are redundant. In both cases, employees feel they should not be expected to do these tasks because they do not respect the rules and norms associated with their professional roles (Semmer et al., 2010, 2015).

Understanding how supervisors’ personality traits influence their choice of participative leadership behavior constitutes an important research task. With this second study we like to investigate how the three facets of multidimensional perfectionism proposed by Hewitt and Flett (1991) are associated with the usage of delegation and participation in supervisors. In the following, specific hypotheses are proposed regarding the relationships of perfectionism facets in supervisors with their delegation and participation behavior.

As self-oriented perfectionism is related to high standards for oneself and an internal drive to achieve perfection, it can be expected to be associated with difficulties in delegation. Since self-oriented perfectionists’ standards are above average, it is likely that a task done by someone else, for instance a subordinate, will not meet these standards (Hewitt & Flett, 1991). Consequently, supervisors high in self-oriented perfectionism should prefer doing tasks themselves to ensure a satisfactory outcome. For the same reasons we expect them to use less participation in general. At the same time, self-oriented perfectionism has been linked to prosocial behaviors and indicators of social connection such as empathy (Stoeber et al., 2017). In previous research on perfectionism and leadership style, this dimension even turned out to be rather favorable concerning servant leadership, especially concerning the subdimension humility that describes the awareness of one’s strengths and weaknesses (Otto et al., 2021). Despite their tendency to do tasks themselves, we also expect supervisors high in self-oriented perfectionism to avoid the assignment of illegitimate tasks, given their general prosocial orientation.

In contrast, socially prescribed perfectionism has shown links to antisocial orientations as well as social disconnection (Sherry et al., 2016; Stoeber et al., 2017). In supervisors, it was shown to be linked to both active as well as passive management by exception, such as immediately reacting to errors or problems (Otto et al., 2021). Therefore, this dimension could also be related to difficulties with delegation and participation. Contrarily, socially prescribed perfectionism is related to avoidance of criticism and failure (Hewitt & Flett, 1991). Because Otto et al. (2017) found delegation to be especially used if a decision is bothersome, delegating tasks and responsibilities to subordinates could be a mechanism for socially prescribed perfectionistic supervisors to evade having to deal with certain tasks and responsibilities themselves. Thus, they would use more delegation. Both mechanisms could be present in supervisors high in socially prescribed perfectionism, as current research shows support for both opposing directions of effects. Therefore, socially prescribed perfectionism will be tested exploratively only.

Finally, given that other-oriented perfectionism is characterized by unrealistically high standards for others, it can be assumed to be negatively related to delegation as well (Hewitt & Flett, 1991). It seems likely that their subordinates’ work procedures or results rarely fully satisfy other-oriented perfectionistic supervisors. Recent findings underline a positive relationship of other-oriented perfectionism and monitoring leadership behavior, i.e., screening the work of their subordinates for any mistakes, irregularities, or deviations from standards (i.e., active management by exception; Otto et al., 2021). Consequently, they might only delegate tasks that do not imply a lot of responsibility and closely monitor their subordinates’ work processes to ensure it meets their demands, thus providing only limited scope of action. They might also care less about employee empowerment and participation, as other-oriented perfectionism is associated with social antagonism and the so called “dark triad” traits (Stoeber, 2014). As stated before, other-oriented perfectionism is negatively linked to agreeableness, a personality trait comprising that is, altruism and cooperativeness (Costa et al., 1991). Regarding the type of tasks supervisors might delegate we speculate other-oriented perfectionistic leaders to be more likely to delegate tasks they perceive as illegitimate and therefore would not want to do themselves.

To conclude, we expect both self-oriented and other-oriented perfectionistic supervisors to have difficulties with delegation and offering participation. However, we also assume that they differ in whether they assign illegitimate tasks, given their different social orientations.

H4: Supervisor self-oriented perfectionism is negatively related to (H4a) delegation behavior as well as to (H4b) offering participation, but also to (H4c) the assignment of illegitimate tasks.

H5: Supervisor other-oriented perfectionism is negatively related to (H5a) delegation behavior and (H5b) offering participation while being (H5c) positively related to the assignment of illegitimate tasks.

### Method

#### Participants

The sample was recruited via social media platforms (Facebook, Xing, SurveyCircle) and a university’s mailing-list. Data collection took place in 2019 using an online survey. There was no compensation offered, participants took part on a voluntary basis and had to respond to all questions to prevent missing data. A total of 183 participants started the questionnaire, of which 35 did not fill out the questionnaire completely and were therefore excluded from further analyses. Two participants were excluded from the analysis because the total time spent was less than 150 seconds which indicates that they might not have taken enough care when reading and answering the questions.

The final sample included 146 supervisors with responsibility for at least two subordinates. Of these, 62 described themselves as female, 83 as male and one as other. The age ranged from 18 to 68 years (*M* = 42.61, *SD* = 13.23). When asked what kind of industry the participants were currently working in, covering most fields from arts and culture over tourism to finances, the most common industry stated was education and science (30.1%). Most participants had already spent more than 10 years in their current position (26.7%). Supervisors were also asked to indicate on how many subordinates they were supervising, response options ranging from “2 subordinates” to “more than 20”. Most participants (26.0%) were responsible for 3 to 5 subordinates followed by those responsible for more than 20 subordinates (21.9%). The number of hierarchy levels below the supervisors was of interest as well, with response options ranging from “one level of subordinates” to “more than 3 levels of subordinates”. Most participants had only one level of subordinates below them (47.9%) followed by supervisors responsible for two levels (32.9%). Finally, participants reported on how often they were in contact with their subordinates, with response options ranging from “not more than once a month” to “every day”. More than half of the participants (58.2%) were in contact with their subordinates on a daily basis, 28.8% at least several times per week.

#### Instruments

##### Perfectionism

Multidimensional perfectionism was investigated in the same way as in the first study with the facets revealing satisfying reliabilities (Nunnally & Bernstein, 1994): self-oriented perfectionism (α = .84), socially prescribed perfectionism (α = .77), and other-oriented perfectionism (α = .74).

##### Participation

Two sub-scales of the German Health- and Development-promoting Leadership scale (GEFA; Vincent-Höper & Stein, 2019) were used to measure leadership behavior that promotes employee career development through participation, i.e., participation itself and scope of action. Both scales showed sufficient individual Cronbach’s alpha values. Participation was assessed with a 4-item scale (e.g., “I include workers in decisions that affect their work or workplace environment.“; α = .84) and scope of action with a 3-item scale (e.g., “I allow the workers to decide for themselves how they organize their tasks.“; α = .80). For both scales, responses ranged from “does not at all apply” (1) to “fully applies” (5).

##### Delegation

Due to a lack of a respective measure, a new questionnaire to identify different aspects of delegation was ad-hoc developed (see, Appendix for the items). Overall, 11 items based on the present literature on delegation as well as the consultation of experts were developed. All items were presented with a 5-point Likert scale, ranging from “never” (1) to “always” (5). Principle Components Analysis method (PCA) with oblique rotation (method: Oblimin; Delta = 0) was applied to all questionnaire items. In accordance with the Kaiser-Meyer-Olkin-Criteria the data was suited for conducting a PCA with a value of .77. A parallel analysis showed two components whose eigenvalues were higher than the ones created from randomly generated data. Therefore, these two components were extracted for further analysis, accounting for 45% of the variance.

The first factor consisted of 6 items with factor loadings ranging from .52 to .77. The items in this subscale relate to the ability to transfer tasks (e.g., “If there is much to do, I hand over some of my tasks to other persons involved”), and we therefore labelled it as ‘delegation of tasks’. This 6-item scale showed a satisfactory reliability with a Cronbach’s alpha value of α = .76.

The second scale consisted of 5 items with factor loadings ranging from .50 to .71. The items in this subscale relate to the ability to hand over responsibility to subordinates (e.g., “If I transfer tasks to other people, they bear responsibility for all steps”) and we therefore called it ‘delegation of responsibility’. This scale did not show a sufficient Cronbach’s alpha with a value of .61. However, the alpha value also depends on the length of the scale and the breadth of the higher order construct. Therefore, it is important to take the inter-item correlations into account, especially for short scales (Streiner, 2003). Mean inter-item correlations between .15 and .20 are suggested for measuring broader constructs and correlations between .40 and .50 for very narrow constructs. The mean inter-item correlation for this subscale accounts for .24. Thus, the scale was included in further analysis allowing the assessment of the construct of delegation from a broader perspective.

Note, to further control for potential empirical overlap regarding the four measures (a) delegation of tasks, (b) delegation of responsibilities, (c) granting participation, and (d) granting scope of action, we conducted a confirmatory factor analysis (CFA). Specifically, we contrasted a single-factor model in which all items were clustered on a global factor (χ^2^ = 336.03, *df* = 132, χ^2^/*df* = 2.55, CFI = .65, RMSEA = .11) with the theoretically proposed four-factor model (χ^2^ = 179.55, *df* = 126, χ^2^/*df* = 1.43, CFI = .91, RMSEA = .06). The four-factor model significantly outperformed the single-factor model, Δχ^2^ = 156.48, df = 6, *p* < .001, and showed overall a satisfactory model fit. Thus, according to the CFA, the four measures appear to represent different underlying constructs.

##### Illegitimate tasks

The distribution of illegitimate tasks by supervisors was assessed with the Bern Illegitimate Task Scale (Semmer et al., 2010). The scale consists of 8 items and participants answered on a 5-point Likert scale, ranging from “never” (1) to “always” (5). As the scale measures the perceived number of illegitimate tasks received by an employee the original item wording was changed to a (self-rated) manager’s perspective to fit the purpose of this study. The scale is divided into two subscales, one referring to the assignment of unnecessary tasks (4 items; e.g., “Do you transfer work tasks to take care of which keep you wondering if they make sense at all?‘) and the second one referring to unreasonable tasks (4 items; e.g., “Do you transfer work tasks to take care of, where you think they go too far, so you can’t really be expected to do it?“). Both subscales showed sufficient internal consistency with a Cronbach’s alpha value of α = .81 for the assignment of unnecessary tasks, and α = .74 for the assignment of unreasonable tasks.

### Results and discussion

As illustrated in Table 4 and in line with Study 1, all three perfectionism scales showed moderate to large-sized positive correlations with each other (*p* < .01).

**Table 4.** Correlations and Descriptive Statistics (Study 2, Supervisors).

Variable	1	2	3	4	5	6	7	8	9	10	11	12
1. Gender												
2. Age	.21*											
3. Supervisory tenure	.31***	.61***										
4. Self-oriented perfectionism	−.03	−.14	−.09									
5. Socially prescribed perfectionism	.10	.06	.17*	.45***								
6. Other-oriented perfectionism	.06	.03	.02	.55***	.50***							
7. Delegation of tasks	.15	.22*	.07	−.42***	−.24**	−.20*						
8. Delegation of responsibilities	−.10	.15	.12	−.29**	−.15	−.27**	.37***					
9. Granting participation	−.12	.08	.01	−.10	−.06	−.09	.27**	.33***				
10. Granting scope of action	−.15	.11	.07	−.25**	−.17	−.11	.37***	.40***	.51***			
11. Assignment of unnecessary tasks	−.12	−.08	−.10	−.09	.13	.08	−.09	−.12	−.02	−.13		
12. Assignment of unreasonable tasks	−.09	−.24**	−.14	.01	.13	.01	−.10	−.09	−.03	−.21*	.38***	
*M*	——	42.61	3.46	4.63	3.27	3.89	3.17	3.25	4.00	4.20	2.22	2.01
*SD*	——	13.23	1.25	1.23	1.14	1.01	0.52	0.51	0.60	0.49	0.68	0.68
Cronbach’s alpha	——	——	——	.84	.77	.74	.76	.61	.84	.80	.81	.74

*Note. N* = 146. For gender, 1 = female, 2 = male. For supervisory tenure, 1 = less than 6 months, 2 = between 6 months and 2 years, 3 = between 2 and 5 years, 4 = between 5 and 10 years, 5 = more than 10 years. Scores are mean scores with a possible range of 1–7 for perfectionism and 1–5 for all other measures with higher values indicating stronger endorsement to the construct.

*p < .05. **p < .01. ***p < .001.

Mirroring our approach in Study 1, we used hierarchical regression analyses to examine the unique relationships of self-oriented, other-oriented, and socially prescribed perfectionism with delegation of tasks and responsibilities, participation, scope of action, and assignment of unnecessary versus unreasonable tasks, respectively. Similar to Study 1, gender, age, and tenure (this time considering supervisory tenure i.e., years of having leadership responsibility instead of professional tenure) were considered as controls. The results are shown in Table 5.

**Table 5.** Hierarchical Regression Analyses: Predicting Career-relevant Delegation Behavior by Supervisors’ Perfectionism (Study 2).

		*B*	*SE*	β		*CI 95 (%)*	*R* ^2^_change_	*B*	*SE*		β	*CI 95 (%)*		*R*^2^change
		Delegation of tasks						Delegation of responsibilities						
Step 1		3.54					.074*	3.84						.043
Gender		0.14	.09	.14		[–.025, .291]		−0.14	.09		−.14	[–.324, .024]		
Age		0.01	.00	.22*		[.001, .018]		0.00	.00		.11	[–.006, .014]		
Supervisory tenure		−0.05	.04	−.13		[–.141, .028]		0.03	.05		.08	[–.062, .132]		
Step 2							.163***							.094 **
Self-oriented perfectionism		−0.16	.04	−.38***		[–.246, −.090]		−0.08	.04		−.18^✝^	[–.158, .004]		
Socially prescribed perfectionism		−0.05	.04	−.19		[–.128, .036]		0.01	.05		.02	[–.088, .115]		
Other-oriented perfectionism		0.03	.05	.06		[–.076, .140]		−0.09	.05		−.18^✝^	[–.202, .026]		
		Granting participation						Granting scope of action						
Step 1		4.46					−026	4.52						.045
Gender		−0.13	.09	−.14		[–.335, .055]		−0.20	.10	−.19*		[–.403, −.017]		
Age		0.01	.01	.12		[–.001, .013]		0.01	.01	.08		[–.007, .014]		
Supervisory tenure		−0.01	.05	−.03		[–.106, .085]		0.03	.05	.07		[–.072, .129]		
Step 2							.009							.062*
Self-oriented perfectionism		−0.03	.05	−.06		[–.124, .073]		−0.10	.05	−.23*		[–.199, −.001]		
Socially prescribed perfectionism		0.01	.05	.01		[–.080, .085]		−0.05	.05	−.10		[–.133, .044]		
Other-oriented perfectionism		−0.03	.06	−.05		[–.151, .096]		0.04	.06	.07		[–.008, .161]		
	Assignment of unnecessary tasks							Assignment of unreasonable tasks						
Step 1	2.71						.018	2.66						.060*
Gender	−0.15		.12	−.11	[–.405, .101]			−0.08	.12	−.06			[–.318, .181]	
Age	−0.01		.91	−.05	[–.015, .010]			−0.01	.01	−.24*			[–.026, .001]	
Supervisory tenure	−0.05		.06	−.10	[–.185, .070]			−0.01	.06	−.02			[–.146, .114]	
Step 2							.069*							.034
Self-oriented perfectionism	−0.15		.06	−.28*	[–.267, −.044]			−0.07	.06	−.12			[–.181, .045]	
Socially prescribed perfectionism	0.13		.06	.22*	[–.021, .266]			0.13	.06	.21*			[.090, .236]	
Other-oriented perfectionism	0.09		.08	.13	[–.076, .255]			−0.01	.08	−.02			[–.164, .148]	

*Note.* For details, see Table 4. CI = confidence interval. Confidence intervals rely on 5000 bias-corrected and accelerated bootstrap-samples.

✝ *p < .10. *p < .05. **p < .01. ***p < .001, two-tailed tests.

Regarding *supervisor self-oriented perfectionism*, we proposed, on the one hand, negative links to delegation behavior and enabling participation, yet on the other, a lower likelihood to assign illegitimate tasks. Results are overall in line with our assumptions. Specifically, self-oriented perfectionism showed a moderate negative correlation with delegation of tasks, and a smaller and only marginally significant negative correlation with the delegation of responsibility, confirming H4a. When it comes to participation in a broader sense, self-oriented perfectionism showed unique negative relationships with enabling scope of action but not with participation, partly supporting our assumptions in H4b.

As self-oriented perfectionists set standards for themselves and for their work that are above average, they might not trust their employees to perform as well as they perform themselves (Hewitt & Flett, 1991). They might find it easier to do tasks themselves to ensure a high-quality outcome. The negative correlations with delegation of tasks as well as delegation of responsibilities indicate that supervisors high on self-oriented perfectionism find it difficult to hand off tasks to subordinates, even if these tasks could easily be done by other people, thus potentially reducing their workload. Also, they seem to struggle to entrust their subordinates with the full responsibility for a task or project, denying them the opportunity to decide process steps on their own and potentially interfering with the results if they do not meet their expectations. Yet, having responsibility is essential for developing skills and competencies important for promotions and career progress. The negative correlation with scope of action further indicate that self-oriented perfectionists also feel the need to have a high amount of control over employees’ daily work routines and face difficulties in letting employees plan and realize their work themselves.

Interestingly, and supporting H4c we also found supervisors high in self-oriented perfectionism to be less likely to assign unnecessary tasks to their employees illuminating also the rather positive side of this perfectionism facet. Illegitimate tasks are associated with impairments of psychological well-being in employees (Semmer et al., 2015) and decreasing job satisfaction (Kottwitz et al., 2021). By not delegating such tasks that are perceived as senseless, self-oriented perfectionistic supervisors contribute to their subordinates’ well-being and insofar do not further damage their career development chances.

In contrast to self- and other-oriented perfectionism, *supervisor socially prescribed perfectionism* was positively related to the assignment of both types of illegitimate tasks. However, although the regression coefficient was significant, the confidence intervals for the assignment of unnecessary tasks showed a somewhat different picture, limiting the robustness of the finding for either of the two outcomes. Nevertheless, the overall pattern suggests that if supervisors are high in socially prescribed perfectionism, they are more likely to pass on tasks to their employees that they perceive as senseless or invalid. Perhaps, they consider mistakes in these tasks as tolerable and try to avoid severe mistakes in more important tasks themselves. Illegitimate tasks are tasks that violate norms about what an employee can legitimately be expected to do inherent in one’s perceived professional role (Semmer et al., 2015, 2019) highlighting the tendencies of social disconnection of socially prescribed perfectionists (Kleszewski & Otto, 2020). The bivariate negative correlation with delegation of tasks did not reach significance in the regression analysis. This result hints that socially prescribed perfectionists might generally show difficulties in sharing tasks with their employees but if they do so these supervisors might only delegate such tasks they perceive as unreasonable or unnecessary.

When it comes to *supervisor other-oriented perfectionism* we expected negative relationships to delegation behavior and granting participation while positive association to the assignment of illegitimate tasks were assumed. However, supervisor other-oriented perfectionism only showed a marginally negative relationship with delegation of responsibility, providing some support for H5a while contradicting H5b and H5c. Other-oriented perfectionists have very high expectations of others that are difficult to fulfill (Hewitt & Flett, 1991). Therefore, they should rarely feel truly satisfied by the work their employees submit when it was delegated to them. Consequently, they rather object handing over tasks. Unique negative correlations for other-oriented perfectionism were obtained for delegation of responsibilities but not for delegation of tasks. These findings imply that other-oriented perfectionistic supervisors do not necessarily find it more difficult to pass on tasks to their employees. However, they might only entrust their employees with tasks of low responsibility. Furthermore, they might supervise employees’ work process and results more closely and tend to interfere frequently to verify that employees meet their high demands (Otto et al., 2021). While findings linking other-oriented perfectionism to antisocial traits (Stoeber, 2014), this facet showed no unique correlations with illegitimate tasks, suggesting that other-oriented perfectionists are not more inclined to delegate tasks they perceive as unnecessary or unreasonable.

## General discussion

With the present work, we aimed at extending the research avenue linking perfectionism, with career choices, career adjustment and career success. Observing the role of multidimensional perfectionism for employees’ career development we set out to conduct two studies enabling us a multi-perspective insight. Drawing on conservation of resources theory (Hobfoll, 1989; Hobfoll et al., 2018), we investigated the role of employee and supervisor perfectionism in shaping career development. Specifically, the first study explored how employee perfectionism is linked to career aspirations (i.e., work motivation) and perceived career success. The aim of the second study though was to investigate the impact of supervisors’ perfectionism on such type of participative leadership behavior that promotes subordinates’ career development, focusing especially on delegation. Overall, our findings demonstrate that each of the dimensions of perfectionism is relevant for employees’ career development.

Self-oriented perfectionism can be described as ambivalent concerning employees’ career development. In Study 1, employee self-oriented perfectionism was positively related to extrinsic material motivation and other-referent career success, highlighting that employees high on this dimension of perfectionism are generally focused on material and career success compared to their peers. In Study 2, supervisors’ self-oriented perfectionism was negatively linked to delegation of tasks and responsibilities, and to granting scope of action. Further, supervisors high in self-oriented perfectionism were less likely to assign unnecessary tasks to their team members. Thus, these supervisors at least not harm their team members’ career development by delegating unnecessary tasks. However, they may neither support their development by assigning responsibility and providing opportunities to acquire new skills and competencies for career progress. Yet, individuals high on self-oriented perfectionism have been found to show overall prosocial behaviors and to care for others (Stoeber, 2014; Stoeber et al., 2017). The supervisors’ tendency to do tasks themselves to ensure a perfect result may, probably without intending it, interfere with their team members career development.

Both employee and supervisor socially prescribed perfectionism, on the contrary, were found to be unfavorable concerning ones’ own and the team members’ career development. In Study 1, employee socially prescribed perfectionism was negatively linked to career satisfaction, other-referent and self-referent career success. From the supervisors’ perspective in Study 2, socially prescribed perfectionism was related to the assignment of unnecessary and unreasonable tasks, which may drain employees’ resources and lead to impaired well-being (Semmer et al., 2015) and decreased job satisfaction (Kottwitz et al., 2021). However, it should be noted that this antisocial tendency is caused by these supervisors’ fear of criticism and failure (Hewitt & Flett, 1991). Paradoxically, the assignment of illegitimate tasks may be seen as an even more serious failure and as falling short of the responsibility of a supervisor.

Lastly, other-oriented perfectionism turned out to be unfavorable with regard to employees’ own career aspirations in Study 1 and supervisors’ willingness to delegate responsibility in Study 2. Specifically, in Study 1, other-oriented perfectionism was not only positively related to extrinsic social motivation and amotivation but also negatively related to intrinsic motivation. Thus, this dimension with its external focus may be described as distracting employees from pursuing their own goals.

In conclusion, this pattern of findings demonstrates that both self-oriented perfectionism and socially prescribed perfectionism as those dimension that are directed towards oneself may not only affect personal outcomes, such as one’s own career progress, but also have consequences for significant others, such as the team members’ career development. Equally, other-oriented perfectionism which is directed towards others and commonly proposed to affect especially these others (e.g., Childs & Stoeber, 2012), can be highly relevant for one’s own aspirations (i.e., motivation). Thus, our findings illustrate the value of integrating all dimensions of perfectionism with their different sources and directions of demands in research questions.

### Strengths, limitations, and future directions

The present research contains some strengths that underlie the important contributions of the paper towards understanding how perfectionism may facilitate or hinder career development of employees. Theoretically, the study provides new insights in the role of personality – i.e., perfectionism – as antecedent of career development. Whereas extant research has mainly focused on the role of perfectionism on career processes and outcomes such as career adaptability, career decision-making, and academic achievement (e.g., Damian et al., 2016; Kundi et al., 2021; Wang et al., 2020), little attention has been paid to the role of perfectionism on various aspects of career success (Kundi, et al., 2021). The present study extends existing literature by demonstrating how the different dimensions of employee and employer perfectionism can thwart or boost career aspirations and subjective career success.

Previous research has either focused on the sum of the perfectionism construct or on individual dimensions. Rarely has research focused on all the three dimensions (self-oriented perfectionism, socially prescribed perfectionism, and other-oriented perfectionism). Therefore, our study is one of the few efforts that have focused on all dimensions in the context of careers. Moreover, the present study considers the three dimensions from the perspectives of both employees and supervisors. By focusing on employee and supervisor samples, we are able to demonstrate how each of the perfectionism dimensions’ thwarts or promotes career progressions either from the employees’ behaviors and the supervisors’ treatment of employees, which is an important contribution to both perfectionism literature and career development literature.

Despite these strengths and the important contributions, the study makes to the perfectionism and career development literature, our findings should be generalized with caution due to a number of weaknesses. First, whereas the paper reports findings from two papers focusing on samples of employees and supervisors, both studies were cross-sectional, hence cannot make concrete causal inferences (Levin, 2006). Given that perfectionism is conceptualized as a personality trait and has been demonstrated to remain comparatively stable over time (e.g., Sherry et al., 2016), a natural causal precedence can be assumed for its associations with the variables of interest. Nevertheless, dyadic studies linking employees with their supervisors are strongly needed.

Second, regarding the samples, Study 1 focused on the specific sample of administrative staff from one university while Study 2 focused on a convenient sample of supervisors of whom the majority worked in the education field. This may limit the generalizability of our results to employees and supervisors in other professional fields. In addition, the sample sizes of both studies could be considered quite small. Post hoc power analyses showed that our samples were adequately powered to detect medium to large effects. However, with a larger sample size, we might have found small to moderate effects (such as the marginally negative relationship between supervisors’ self- and other-oriented perfectionism and delegation of responsibility to subordinates, see Table 5). Future research should examine larger samples that include employees and supervisors from different occupational backgrounds over time.

Third, when evaluating the selected outcomes, in Study 1, perfectionism dimensions were differentially associated to the different forms of motivation. However, we only examined the facets of amotivation, extrinsic motivation (facets: social and material), and intrinsic motivation - while neglecting the facets of for identified and introjected motivation (Deci & Ryan, 1985; Ryan & Deci, 2000). We recommend that future studies should comprehensively examine motivation as an underlying mechanism through which perfectionism influences career success. In Study 2, in contrast, due to the lack of an appropriate measure of delegation, we developed a questionnaire to identify various aspects of delegation, albeit in an ad hoc manner. While this questionnaire (see Appendix) was found to be valid and reliable for the present study, researchers should use it cautiously in future studies. Therefore, there is a strong need to develop a delegation measure to support future research.

Last, the paper discusses the role of employee and supervisor perfectionism in career success. However, across the two studies, we focused on career success of employees but not that of the supervisors, which could also be important to establish. Moreover, the studies predominantly focused on indicators of subjective career success only, employee and supervisor perfectionism could be equally important in explaining objective career success. Future research should entangle the role of supervisors’ own career interests when it comes to the effects of their perfectionism scores on the career development of their subordinates. Assuming that those high in self-oriented perfectionism pursue career goals and want to climb the ladder they might on the same hand not be willing to let others (i.e. their subordinated employees) spoil the water.

### Practical implications

The paper contributes to the understanding of career success and the essentiality of perfectionism in the process of attaining specifically subjective career success. Kundi et al. (2021) outlines why it is important to understand the underlying factors for subjective career success both for individuals and organizations. Beyond the contributions the study makes to perfectionism and career development literature, our study has implications for practice. We particularly present implications for individual employees and for employers (supervisors).

For employees, our findings suggest that one’s own perfectionism tendencies can boost or thwart career development and success. It therefore important that employees develop awareness of own perfectionistic tendencies and the lively consequences. This can enable one to be cautious of certain practices that are likely to injure one’s careers progress and success given that self-awareness is essential in exercise of self-control (Alberts et al., 2011). Career counselors can support employees in exploring their perfectionistic behaviors, likely career outcomes, and ways of exercising control, for example, using guided self-help (Stoeber & Damian, 2016).

Similarly, particularly results of the Study 2 show that the perfectionistic tendencies of supervisors also have potential to bolster or derail employees’ career development and success by providing or draining important resources. It is important for supervisors to be aware of the nature of their own perfectionistic orientation and its consequences not only on their own career progress and success but on the career progress and success of their subordinates. Knowing that one’s own behavior will impact negatively on the careers of those one is meant to nurture can be helpful step for supervisors to adopt behaviors that promote development of skills and professional identity and to drop or control supervisor behavior that circumvents subordinates career development.

### Conclusion

This paper contributes to the growing discourse on the role of perfectionism in career development by particularly focusing on the association between the three dimensions of perfectionism dimensions (i.e. self-oriented perfectionism, socially prescribed perfectionism, and other-oriented perfectionism) and the different indicators of subjective career success from the employee (work motivation and subjective career success) and supervisor perspectives (delegation and employee participation). Our findings suggest that both employee and supervisor perfectionism have potential to boost or thwart employee career development and success in different ways. Specifically, whereas employee self-oriented perfectionism boosts career success reflected in the aspiration for extrinsic material rewards, supervisor self-oriented perfectionism may thwart employees’ career development and success due to reluctance to delegate tasks and responsibility that could foster skill development and mastery among employees. On the other hand, employee socially prescribed perfectionism tends to obstruct career development and success since it was observed to negatively correlate with career success indicators. Moreover, supervisor socially prescribed perfectionism could be seriously detrimental to career success given that such supervisors tend to delegate illegitimate tasks to their subordinates. Finally, employee other-oriented perfectionism could undermine one’s career development and success because it tends to lower intrinsic motivation while at the same time heighten extrinsic social motivation hence the diversion from pursuing one’s own career aspirations. Not surprisingly, supervisors high in other-oriented perfectionism may not delegate tasks of very high responsibility. However, reluctance to delegate high responsibility tasks to subordinates can thwart career development and success since it may limit opportunities for experiential learning. Against the backdrop of our findings, job applicants and employees applying for a leadership position may consider new answers to the question about their biggest weakness.

## Data Availability

The data that support the findings of this study are available from the corresponding author upon reasonable request.

## Appendix

Ad-hoc developed items measuring delegation behavior

(Note that the items were originally developed in German and administered to a German-speaking sample. Items 2, 3, 5, and 6 of the Delegation of Tasks subscale and items 3, 4, and 5 of the Delegation of Responsibility subscale must be reversed.)

### Delegation of Tasks

1. If there is much to do, I hand over some of my tasks to other persons involved.
2. I prefer to do important tasks myself so that they meet my requirements. (−)
3. Handing over tasks is rarely worth it for me. (−)
4. I also give tasks to other people that will challenge them so that they can develop further.
5. I often do tasks that could also be done by other people involved. (−)
6. Tasks that I have given to someone else, I end up doing myself. (−)

### Delegation of Responsibility

1. If I transfer tasks to other people, they bear responsibility for all steps.
2. When I hand over tasks to other people, they are given the opportunity to decide for themselves how they want to proceed.
3. If a person has not completed a task the way I imagined it, I change the result according to my ideas. (−)
4. When I hand over tasks to other people, I expect them to be carried out exactly as I had imagined. (−)
5. When I hand over tasks to other people, I check the results very carefully afterwards. (−)
